# Enneagram typologies and healthy personality to psychosocial stress: A network approach

**DOI:** 10.3389/fpsyg.2022.1051271

**Published:** 2022-11-24

**Authors:** Cristian Ramos-Vera, Antonio Serpa Barrientos, Jonatan Baños-Chaparro, José Vallejos Saldarriaga, Jacksaint Saintila

**Affiliations:** ^1^Research Area, Health Science, Universidad Cesar Vallejo, Lima, Peru; ^2^Peruvian Society of Psychometry, Lima, Peru; ^3^Facultad de Psicología, Universidad Nacional Mayor de San Marcos, Lima, Peru; ^4^Escuela de Medicina Humana, Universidad Señor de Sipán, Chiclayo, Peru

**Keywords:** personality, Enneagram, psychosocial stress, network analysis, personality styles, university students

## Abstract

**Introduction:**

Enneagram typologies may impact psychological well-being and stressful situations in college students. However, the literature is still limited in the study of dynamic personality models such as the Enneagram in Spanish-speaking university students, and a better understanding is needed.

**Objective:**

To analyze network associations and centrality measures of Enneagram personality typologies in Peruvian university students.

**Methods:**

A total of 859 Peruvian university students responded to two instruments assessing: The Pangrazzi’s Enneagram personality types and healthy personality to psychosocial stress. All instruments showed good psychometric values (validity and consistency). A regularized cross-sectional network structure was estimated with Gaussian graphical model and the graphical LASSO.

**Results:**

Enneagram types 4, 5, and 6 presented the highest and positive associations in the network structure. Type 6 emerged as the node with the highest predictability. The healthy personality and type 7 acted as bridges between the communities, with types 6, 7, and 8 being the most central nodes.

**Conclusion:**

The findings suggest that Enneagram type 7 with healthy personality to psychosocial stress plays an important role in the development of the causal activation of the network model. The network shows causal associations between psychosocial stress and types 6, 7, 8, and 9.

## Introduction

Networks allow to represent complex behaviors and phenomena based on a set of interacting variables. Network models are represented by nodes (variables) and edges connecting the nodes representing mutual associations in the network system (Hevey, 2018; Ramos-Vera, 2021). It is possible to assess the complexity of psychological phenomena using these network models that consider patterns of individual differences in normal or psychopathological personality traits (Costantini et al., 2019). Personality models can be identified as an ecosystem in which some characteristics, behaviors, domains, or facets interact with each other, while other connections are reduced given their degree of importance in the network, this allows structuring a unique causal system composed of associations between such personality measures (Costantini et al., 2019).

Personality research has included several models in general populations where well-known personality systems of major research, such as the Big Five or the Hexaco stand out (Thielmann et al., 2021). However, it is important to evaluate other models that have gained less interest to foster important new insights in the assessment of dynamic personality processes (Kuper et al., 2021). The generation of new findings is less likely in the face of the exclusive use of the Big Five model (Mõttus et al., 2020).

One of the least researched personality systems is the Enneagram model that provides insight into psychological structure based on 9 character orientations (types) that include traits, dispositions, and behaviors that are unique to the individual (Alexander and Schnipke, 2020). In this system, each personality type has characteristics based on an underlying motivation grounded in ego responses to a core fear and desire. Individuals may develop a predominant type with other positive and negative characteristics of their Enneagram typological style. However, traits of other typologies are also present in the face of stress and security events that, depend on lifestyle, tend to psychological development, or conversely produce potentially pathological psychological distress (Sutton et al., 2013). This model can help people to understand the mechanisms involved in their own and others’ personality, as well as to know better the dominant styles in social and coping situations involving emotional, cognitive, motivational, volitional, and value aspects.

In the research of Roh et al. (Roh et al., 2019), the different personality styles of the Enneagram (typological triads) are reported as the three basic centers of the human psyche: feeling (types 2, 3, and 4), thinking (types 5, 6 and 7), and instinct (types 1, 8, and 9). Harmonic groups are characterized by the way the person faces and react to disappointment, frustration, or when he/she does not obtain what he/she desires, conforming by positive (types 2, 7, and 9), competitive (types 1, 3, and 5) and reactive (types 6, 4, and 8) styles. They have also been classified according to interpersonal tendencies oriented to the pursuit of needs and desires as referred by psychologist Karen Horney (Hornevian groups), according to the most prevalent typology of the individual, which are the combative style (types 3, 7, and 8), reserved (types 4, 5, and 9), and obedient (types 1, 2, and 6; Roh et al., 2019).

A higher prevalence of negative traits of the dominant personality in stressful situations strengthens the usual patterns of maladaptive coping with emotional distress, which are conceptually similar to the maladaptive schemas referred to in Cognitive-Behavioral Therapy models (Beck, 2013). Therefore, it is likely that other negative traits of other typologies may be manifested that reinforce personality patterns of greater psychological vulnerability, while people with a higher prevalence of positive traits in their typological centers tend to have better psychological development and functioning. In this context, the most important feature that distinguishes the Enneagram from other personality models is the representation of a dynamic system (Enneagram, 2019; Matise, 2019).

Hook et al. (2021) state that the theorization of Enneagram typologies is more closely linked to modern psychodynamic approaches, characterized by the identification of inflexible patterns conducive to emotional experiences aimed at learning new alternatives for social interaction with others. People have been considered to have a cyclical maladaptive pattern (CMP) or a primary pattern of problematic relating in more recent psychotherapeutic models such as Time-Limited Dynamic Psychotherapy. PCM describes patterns of feelings toward the self, expectations and perceptions of others, and ways of relating that are dynamically interconnected and perpetuate dysfunctional relationships (Levenson, 2012). This functioning can also be explained by an underlying motivation rooted in ego responses to a core fear and desire as referenced by the Enneagram model (Hook et al., 2021).

A brief review of the scientific literature on the Enneagram found that most of the studies demonstrating positive effects on college students were conducted in Asia. The little empirical evidence on the integrity and legitimacy of the Enneagram measure in Latin American practice is due to the lack of research in Spanish-speaking participants; however, more research is reported in the United States in diverse areas beyond the university setting (Hook et al., 2021). During the last decade, there has been an increase in studies evidencing the benefits of the Enneagram in the family and work area in various cultural-religious contexts such as Brazil, Spain, Iran, Kenya, United Kingdom, South Africa, and Thailand (Sutton et al., 2013; Burger and van Coller-Peter, 2019; Ndirangu et al., 2019; Navabifar et al., 2020; Romero et al., 2020; Engelseth et al., 2021; Henrique et al., 2021).

Through the theoretical foundation of the Enneagram proposed to the psychological field by the Chilean psychiatrist Claudio Naranjo in 1990 (Naranjo, 1990), several studies have shown its importance in the integration of psychotherapeutic processes, as it strengthens the therapeutic alliance, helps to manage physical and emotional pain, and motivates people to take control in their recovery process (La, 2014; Matise, 2019; Kam and Vriend, 2021). According to the Enneagram model, a better understanding of the mechanisms involved in personality allows people to promote a higher degree of self-compassion and self-acceptance from a greater awareness of the psychological states of imbalance and balance of the most predominant typology; this can motivate individuals to free themselves from their maladaptive schemas, dysfunctional cycles, and limiting defensive styles (Hook et al., 2021).

Some personality types have been reported, which use certain coping strategies in certain contexts, such as the Grossarth-Maticek and Eysenck (Grossarth-Maticek and Eysenck, 1990) model, which refers to the existence of six types of reactions to psychosocial stress that are associated with the presence of symptoms and health-related behaviors such as nutrition, physical exercise, self-medication, or frequency of visits to the doctor (Hernández et al., 2007). Specifically, of interest for the present study is type 4 (healthy personality), similar to type B personality (Shaw and Dimsdale, 2007). Individuals with these personality patterns report a lower degree of emotional reaction of stress and anger to social situations of criticism and competition (Jarašiūnaitė and Perminas, 2014) and a better quality of interpersonal relationships (Kyeum Lee and Jong, 2021). Individuals who identify with other Grossarth-Maticek and Eysenck personalities or types A or D present greater psychological vulnerability (Chilicka et al., 2020; Sakitri, 2020; Fatrous, 2021) and addiction to tobacco use (Mortazavi et al., 2020).

Grossarth-Maticek and Eysenck’s (Grossarth-Maticek and Eysenck, 1990) healthy personality is based on emotional autonomy associated with a higher degree of self-regulation and psychological flexibility (Kirk and Martin, 1998). Individuals identified with a higher predominance of this personality realistically cope with approach and avoidance behaviors with respect to a given stressful event. In addition, they present assertive emotional and behavioral reactions that are socially desirable, such as tolerance, extreme patience, understanding, kindness, and stoic acceptance of problems (Hisam et al., 2014). This personality has been reported to be negatively associated with stress, negative affectivity, aggression, and somatic symptomatology in Spanish adults (Reyes del Paso and Martínez, 2004).

It is important to consider this favorable measure for mental health and physical well-being, verified by the evidence of a higher prevalence of this positive personality to psychosocial stress in young adults in Spain, United States, Norway, and Peru (Sandin et al., 1992; Smedslund, 1995; Martínez-Correa and Reyes Del Paso, 2007; Condori, 2013; Taller, 2018; Núñez, 2020) in contrast to other personality factors with a greater tendency to symptoms of emotional distress and psychosomatic risk (Sandin et al., 1992; Hernández et al., 2007).

The joint evaluation of the network relationships of Enneagram typologies with healthy personality to psychosocial stress reactions allows exploring new findings on the role of variables in the relationship and activation of connections in the network in a systemic way (Epskamp and Fried, 2018; Ramos-Vera, 2021). This allows to know the associative patterns that identify those styles, states, and personality profiles are more influential according to the Enneagram theorization represented in a multivariate network, and to know which ones are more associated with healthy personality (Matise, 2019). Therefore, the present study aims to evaluate network associations and report centrality measures of such personality typologies in male and female university students in Peru.

## Materials and methods

### Participants

The study sample consisted of 859 university students from a private university in the Peruvian city of Ica, who were selected by non-probabilistic convenience sampling. Those university students over 18 years of age, who were registered at the university regular cycle and accepted the informed consent form, were considered. Students who did not meet the inclusion criteria did not participate in the research. In that sense, the final sample was composed of 598 females (69.6%) and 261 males (30.4%), aged 18 to 37 years (Mean: 23.49; Standard deviation: 2.9). The highest percentage of students were from the professional careers of psychology (28.2%) and environmental engineering (26.3%), followed by obstetrics (24.9%) and nursing (20.5%).

### Measures

All measures showed good internal consistency in different Peruvian university groups with adequate psychometric evidence (Vicuña et al., 2001; Condori, 2013; Núñez, 2020).

### Personality according to the Enneagram

Pangrazzi’s (Pangrazzi, 1997) Enneagram questionnaire was used, composed of 9 enneatypes with dichotomous responses (0 = No and 1 = Yes). The enneatypes included 20 items each, which were type 1: Perfectionist (items 1 to 20, for example, “I have an instinctive tendency to evaluate situations”), type 2: Helper (items 21 to 40, for example, “Many people depend on my help and my generosity”), type 3: Accomplishing (items 41 to 60, e.g., “I have a very high energy level”), type 4: Romantic (items 61 to 80, e.g., “I appreciate the beauty of life more than most people”), type 5: Observant (items 81 to 100, e.g., “I generally hide my feelings”), type 6: Loyal (items 100 to 120, e.g., “Fundamentally I am a fairly balanced person”), type 7: Adventurous (items 121 to 140, e.g., “I am the type of person who likes to try a little bit of everything in life”), type 8: Challenger (items 141 to 160, e.g., “I feel able to take a stand and fight for what I believe in”), and type 9: Pacifist (items 161 to 180, e.g., “by nature I am calm, quiet and conciliatory”). The Kuder–Richardson 20 coefficients between the enneatypes were between 0.84 and 0.86, which show adequate values of internal consistency.

### Healthy personality to psychosocial stress

The type 4 personality measure (healthy personality) of the Short Interpersonal Reactions Inventory (SIRI) by Grossarth-Maticek and Eysenck was considered (Grossarth-Maticek and Eysenck, 1990). It contains 10 items (items 4, 11, 18, 25, 32, 39, 46, 53, 60, and 67) with dichotomous response (0 = No and 1 = Yes). This personality measure reported an internal consistency of 0.78 according to the Kuder–Richardson coefficient 20. The Spanish version of Martínez-Correa and Reyes was used, which has adequate psychometric properties (Martínez-Correa and Reyes Del Paso, 2007).

### Procedures

Permission was requested from the director of the university center with the respective information on the purpose of the research and academic purposes. He agreed to carry out the project and provided information to the administrative staff of each faculty to facilitate coordination with the tutor, teachers, and students. The collection of information was carried out during the last 3 months of 2019, in the academic period of cycle II during the tutoring courses in charge of one of the researchers with the support of the tutor in charge.

During the application, each student was explained the objective of the current research and the objectives of the study, and the confidentiality of the participants, who responded voluntarily and anonymously to the survey for an average time of approximately 30 min. Likewise, all procedures used in this study guarantee the confidentiality of the responses and are in accordance with the ethical requirements of the research ethics committee given article 27 of the professional code of Ethics of the Peruvian College of Psychologists and the Helsinki Declaration of 1964.

### Statistical analysis

The Gaussian graphical model (GGM) performed presents a regularized partial correlation network to model the interaction between different variables or psychological phenomena. In this graph, each variable is represented as circles, called “nodes” (or “vertices”). These are connected by lines, called “edges.” In this network variant, the conditional dependency relationships between the variables are characterized: if two variables are connected in the resulting network, they are dependent after adjusting for all other variables. The graphical LASSO (selection operator and absolute minimum shrinkage) was used to estimate the GGM (Epskamp and Fried, 2018) and avoid spurious edges, representing a sparse network describing the data with parsimony. The Fruchterman-Reingold algorithm was used for network visualization, which allows determining the position of a node based on the sum of connections it has with other nodes using the qgraph package (Epskamp et al., 2012). In addition, this model is characterized by coming from a normal distribution, understanding that a pair of random variables comply with the joint normal distribution, on the basis of which it is assumed that their marginal and conditional distribution is also normal.

The precision of the edge weights at 95% confidence intervals was estimated by Bootstrapping 1,000 samples around each edge in the network. The 1,000-sample Bootstrap method was considered to strengthen the stability of the network results; moreover, the strength stability was estimated by calculating the correlation stability coefficient (CS), where the value should not be less than 0.25 and preferably greater than.50 (Fruchterman and Reingold, 1991; Mcnally, 2021). We report the measures of frequency centrality and magnitude of connections that each node has from the number of connections (strength centrality), which have been reported in previous studies (Ramos-Vera et al., 2022).

To identify the bridging variables, the strength-bridge index was considered in the network model, in which Enneagram typologies are connected to healthy personality. Considering Jones et al. (Jones et al., 2021), those variables of interest with the highest bridging centrality were selected based on the percentile parameter >0.80. Such centrality measures have been reported in personality-oriented network research (Goh et al., 2020; Jordan et al., 2021).

## Results

**Figure 1:**
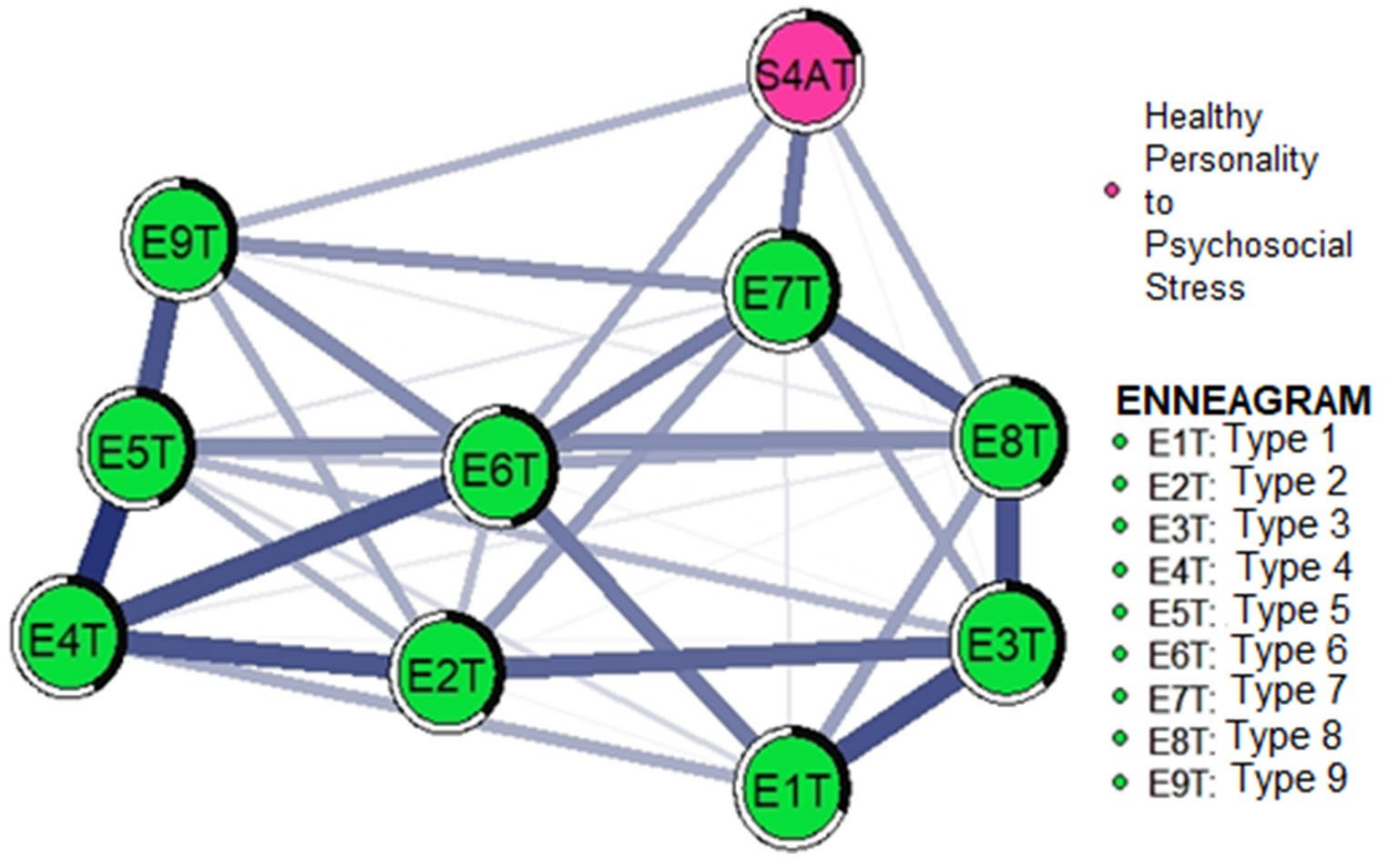
Network analysis of the nine personality types of the Enneagram and healthy personality to psychosocial stress. The greater the thickness of the observed connections, the greater the magnitude of the statistical relationships. It should be specified that the thickness of the line equals the magnitude of the relationship.

Table 1 shows the descriptive statistics of the participants’ responses to the measures used. In the predictability values, the network mean was 37.4%, with type 6 (46.3%) having the highest predictability, followed by type 4 (45.9%), type 5 (45.9%), and type 7 (41.2%). All network structures presented positive correlations, being type 4 with type 5 (0.24) and type 6 (0.21) the highest network associations. Covariances between HP with type 7 (0.16), type 6 (0.11), and type 8 (0.10) were also evident (Table 1). Likewise, in the network graph, the thickness of the connection is evidenced by the magnitude of the correlation, and the shaded proportions of the rings represent the degree of predictability variance between the network nodes (Figure 1).

**Table 1:** Descriptive data, predictability, and network relationships.

Variable	M	SD	P	T1	T2	T3	T4	T5	T6	T7	T8	T9	HP
T1	13.82	3.41	31.50%	-									
T2	12.49	3.59	37.60%	0.01	-								
T3	13.27	3.39	37.80%	0.21	0.18	-							
T4	12.54	3.66	41.90%	0.10	0.20	0.01	-						
T5	12.49	3.45	43.10%	0.05	0.09	0.09	0.24	-					
T6	13.63	3.19	46.30%	0.16	0.08	0.02	0.21	0.07	-				
T7	13.61	3.23	41.30%	0.03	0.12	0.11	0	0.03	0.15	-			
T8	13.39	3.23	40.20%	0.11	0.01	0.2	0.02	0.13	0.11	0.18	-		
T9	13.08	3.48	35.20%	0	0.10	0	0.08	0.20	0.14	0.13	0.02	-	
HP	6.53	2.03	20.20%	0	0	0.01	0	0	0.11	0.16	0.10	0.09	-

M: Media; SD: standard deviation; P: predictability; The T_i values correspond to the partial correlation coefficients. T1: type 1; T2: type 2; T3: type 3; T4: type 4; T5: type 5; T6: type 6; T7: type 7; T8: type 8; T9: type 9; HP: healthy personality.

**Figure 2:**
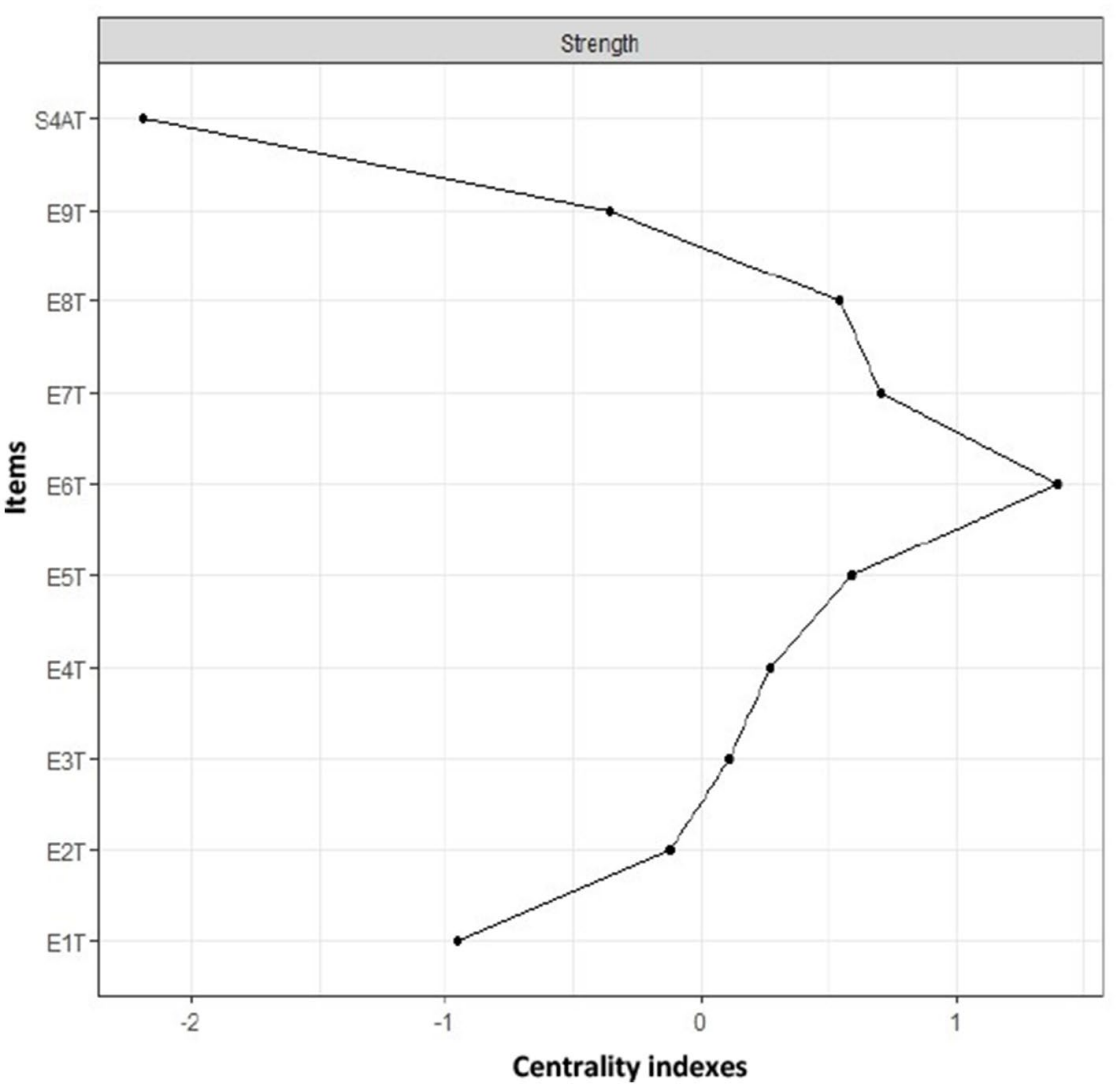
Network analysis strength centrality indexes of network analysis. Centrality refers to the measure with the highest number of connections along with the sum of the relationships it presents. T1: type 1; T2: type 2; T3: type 3; T4: type 4; T5: type 5; T6: type 6; T7: type 7; T8: type 8; T9: type 9; S4AT: healthy personality to psychosocial stress.

Figure 2 shows the measures of strength centrality, where type 6 has a greater influence; other measures of greater centrality were types 7, 8, and 5.

**Figure 3:**
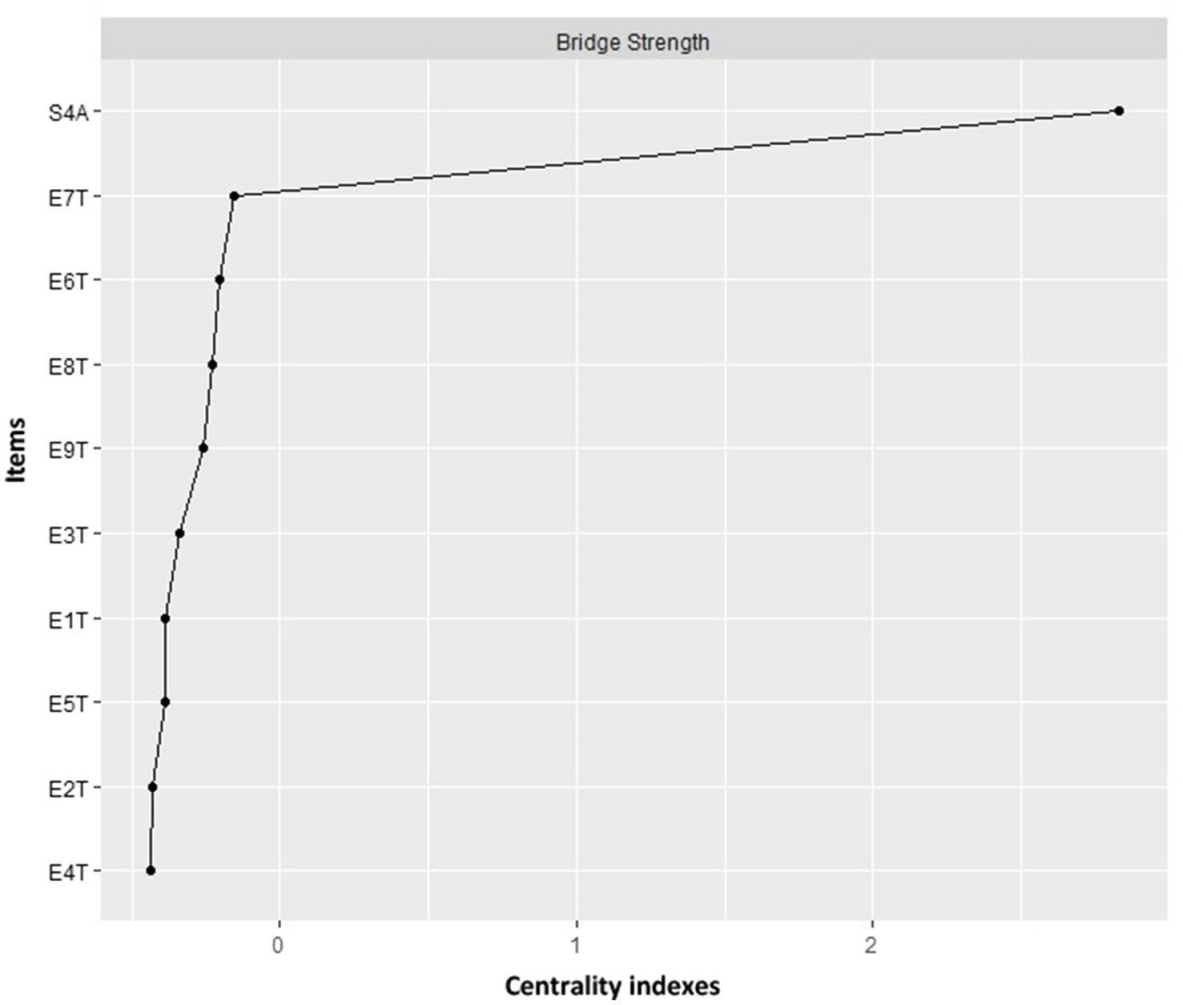
Network analysis bridge strength centrality index. Centrality refers to the measure with the highest number of connections together with the sum of the relationships it presents. T1: type 1; T2: type 2; T3: type 3; T4: type 4; T5: type 5; T6: type 6; T7: type 7; T8: type 8; T9: type 9; S4AT: healthy personality to psychosocial stress.

Figure 3 refers to the bridge strength centrality measures where those greater than 0.80 percentiles are considered, which were healthy personality and type 7, these measures being the ones that play an important role in the development of causal activation of the Enneagram typologies with healthy personality.

**Figure 4:**
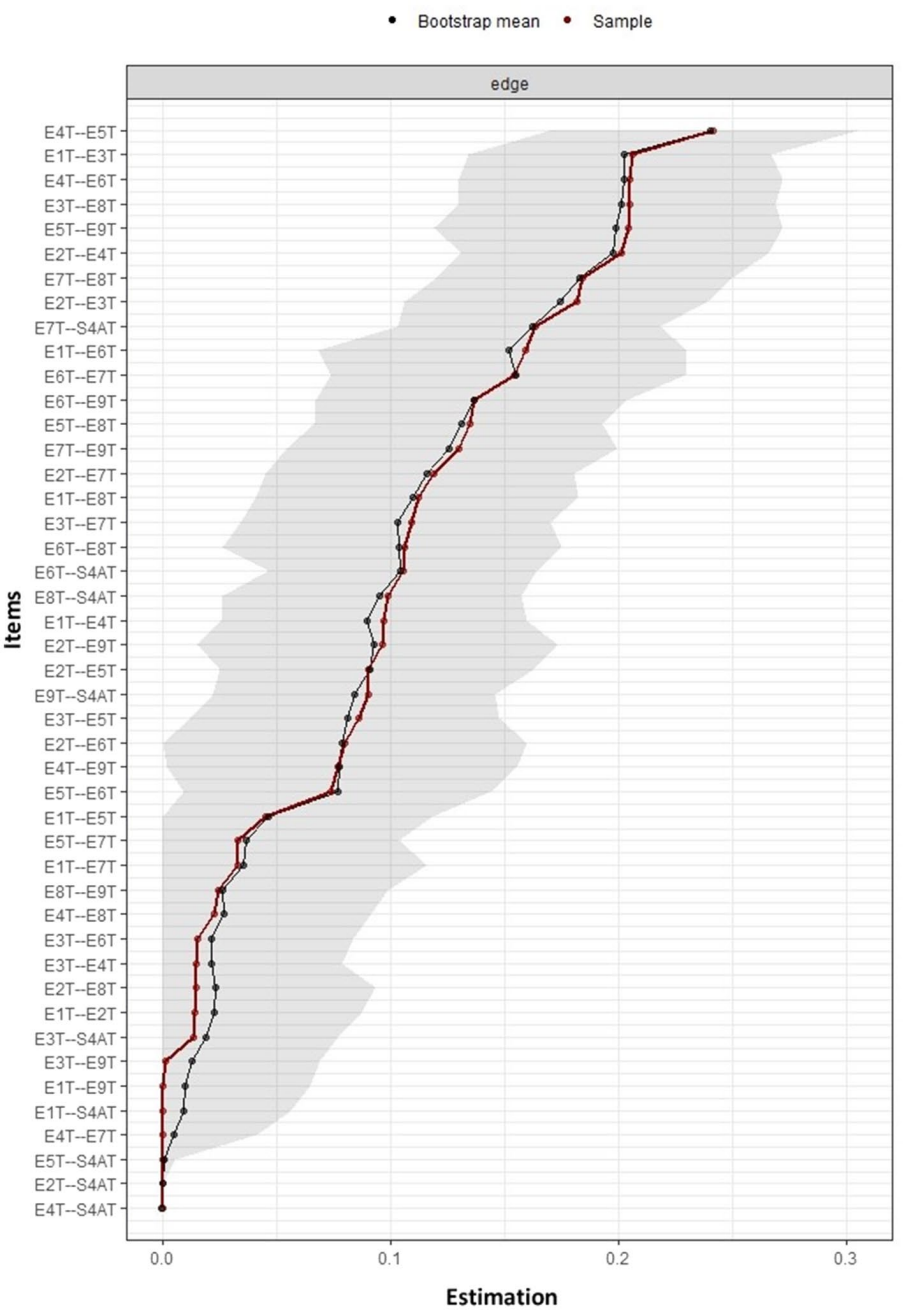
Accuracy of edge weight estimation and 95% CIs based on the Bootstrapping method. The precision of the edge weights is shown, where the red line indicates the sample edge weight (ordered in increasing order) and the gray bars are the 95% CIs based on the Bootstrapping method. T1: type 1; T2: type 2; T3: type 3; T4: type 4; T5: type 5; T6: type 6; T7: type 7; T8: type 8; T9: type 9; S4AT: healthy personality to psychosocial stress.

The precision of the edge weights is shown in Figure 4. It is evident that most of the estimated edges were greater than zero and in general, did not overlap with other edges, reflecting a precise estimation.

**Figure 5:**
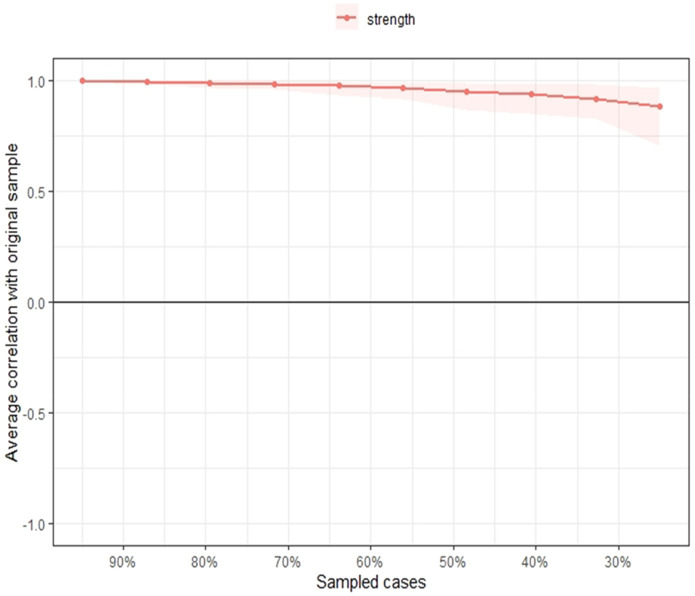
Stability of the strength centrality index. The stability of the strength centrality index is shown, where the red line is the correlation between the strength index estimate and the subsamples that would be used from the total sample.

The stability of the strength centrality index is presented in Figure 5. In that sense, it is observed that the strength estimate is maintained even after removing large proportions of the sample and the CS coefficient showed a value of.71, indicating the stability of the strength of the nodes.

**Figure 6:**
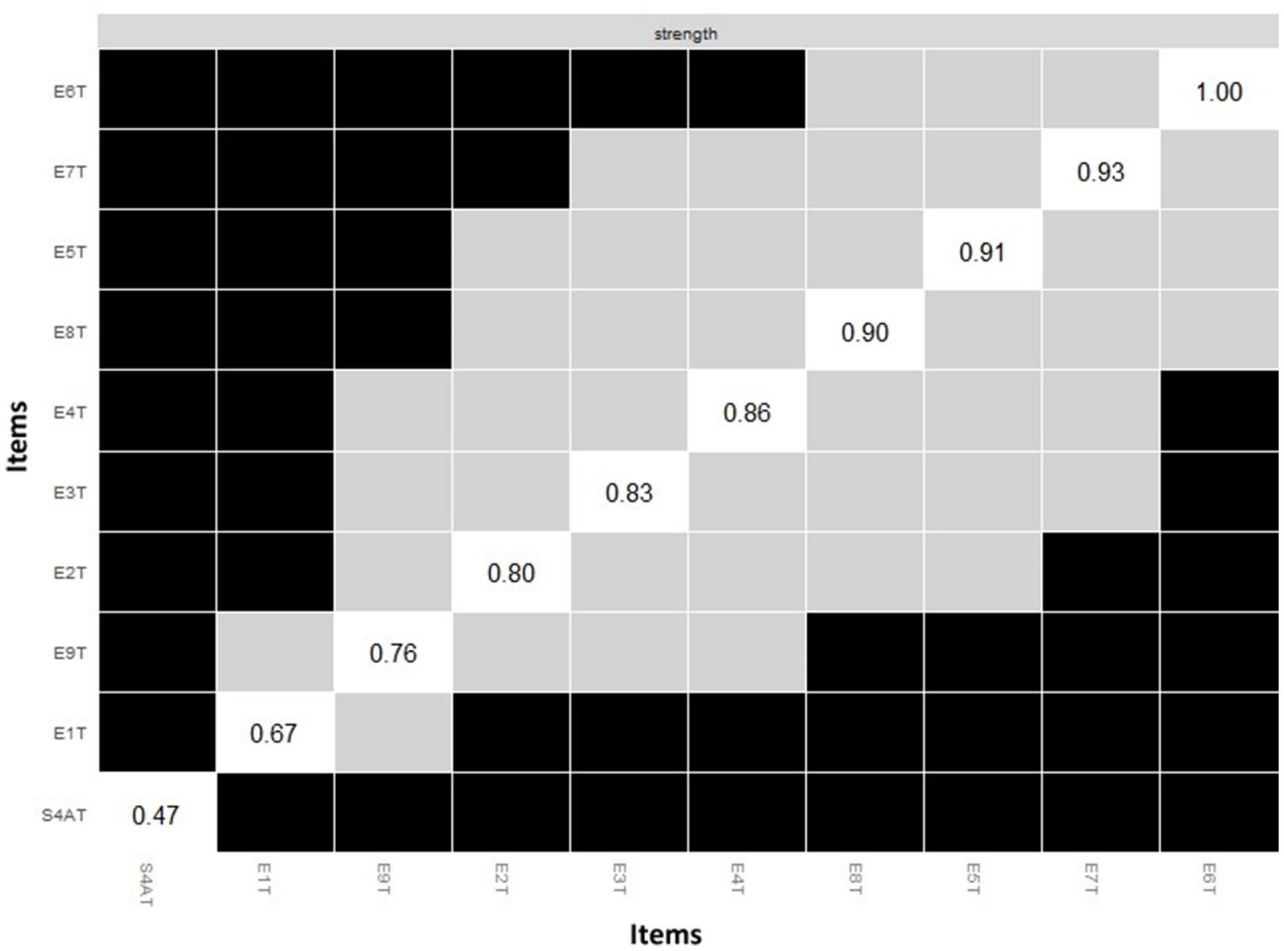
Bootstrap difference test for the strength of nodes. Bootstrap difference test for node strength is evident, where gray boxes indicate non-significant differences and black boxes indicate significant differences. T1: type 1; T2: type 2; T3: type 3; T4: type 4; T5: type 5; T6: type 6; T7: type 7; T8: type 8; T9: type 9; S4AT: healthy personality to psychosocial stress.

The Bootstrap difference test for node strength is presented in Figure 6. This result refers that the healthy personality measure of psychosocial stress was significantly different from all other strength values, while types 1, 9, and 2 were significantly different from most Enneagram typologies in the network.

**Figure 7:**
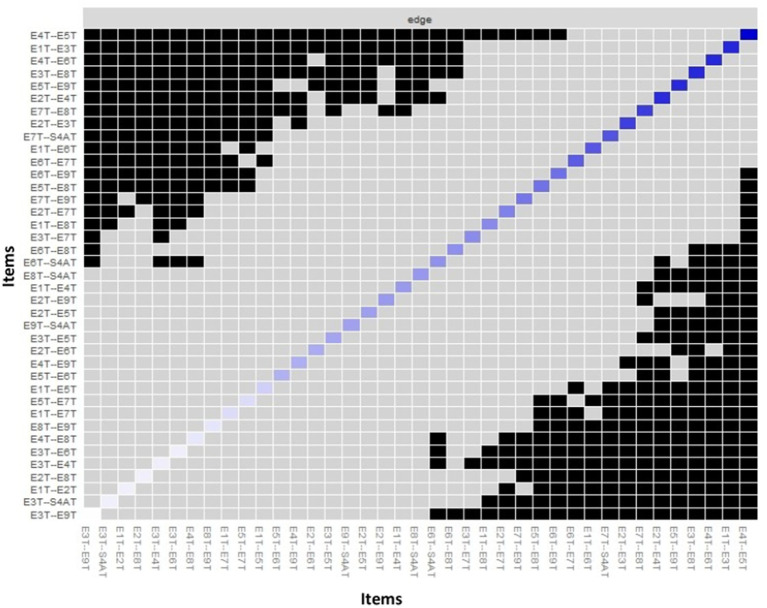
Bootstrap difference test for edge weights. T1: type 1; T2: type 2; T3: type 3; T4: type 4; T5: type 5; T6: type 6; T7: type 7; T8: type 8; T9: type 9; S4AT: healthy personality to psychosocial stress.

Figure 7 presents the Bootstrap difference test for edge weights based on the 95% Bootstrap interval, where the differences of any two edges can include a zero value (dark squares) or not (gray squares), which allows determining whether the two edges are different from each other. The diagonal displays the magnitude of the original edge, where blue squares are positive values and red squares are negative edges, and the color saturation indicates absolute values (the more saturated the color, the stronger the edge). The edge weights between nodes type 4 and type 5, type 1 and type 3, type 4 and type 6, type 3, and type 8 are significantly different from most edges in the network.

## Discussion

The network results refer causal associations of positive reaction to psychosocial stress with types 6 (loyal), 7 (adventurous), 8 (challenging), and 9 (peacemaker), which evidences that university students with healthy behaviors to interpersonal stress may be identified with a more trustworthy, responsible, decisive, outgoing, enthusiastic, innovative, optimistic, and peaceful character. Such typologies are related to higher healthy and assertive behavior (Wagner, 2012), better self-control and positive emotion management (Araghi et al., 2015), and reduced stress (Karabulut et al., 2021) and fear (Al-Obaidi, 2021). Other findings indicate that the healthy personality of lower stressor reactivity is associated with a higher Myers-Briggs perceptual temperament, which is characterized by a high degree of quietness, self-control, and adaptation to new situations (Fretwell et al., 2013).

Enneatype 6 (loyal) is the most influential measure (high degree of strength centrality and predictability) on the other Enneagram typologies and styles in the network, which is characterized by a personality composed of prosocial and emotional traits that contribute to resilience strategies, linked to problem solving. In addition, according to the systemic theory of the Enneagram, it is possible to identify the integration of type 6 with type 9 (peacemaker), which may be an indicator of a better mental health status (Enneagram, 2019), given that both types refer to a network causal relationship and are associated with healthy personality to psychosocial stress. The assessed university students are likely to present a calmer, more mature, and emotionally stable character in the face of interpersonal situations. People who present a personality with greater characteristics of both typological states may have greater control of their psychological needs and efficiently manage anxiety and anger in conflictive social interactions, as referred to in the Enneagram theorizing. This is similar to the development of growth potential and well-being following the satisfaction of basic psychological needs according to self-determination theory (Vansteenkiste and Ryan, 2013) including basic humanistic aspects related to motivation and personality (DeRobertis and Bland, 2018). Individuals with a core personality characterized by type 6 can achieve greater satisfaction of their desire for security and trust when they identify with peaceful personality behaviors (type 9) such as kindness, psychological flexibility, sociability, and empathic listening to others that make it easier for them to cope with distressing interpersonal situations (Hook et al., 2021). These characteristics are related to the Big Five personality facets of extraversion and openness.

Given the greater influence of type 6 on the associative activation of other traits of the intellectual center style (types 5, 6, and 7), iit is probable that cognitive abilities such as intellectual curiosity and openness, critical and analytical thinking are more prelevant (Hook et al., 2021; Kam, 2019). They would promote legislative, judicial, global, hierarchical, and liberal thinking styles that are considered the most adaptive and refer to a complex mode of information processing, as well as a preference for unstructured and holistic situations. This allows individuals to effectively implement their intelligence and creativity in various domains, favoring a high level of self-confidence and autonomy to make decisions and solve problem situations independently (Zhang, 2010). Curiosity is one of the most characteristic traits in people with greater mastery of the intellectual center that strengthens social and intellectual interest, and can even be a motivating force for academic learning (Lauriola et al., 2015). Likewise, people with high levels of motivation and in the face of new experiences report higher degrees of positive emotions and acquisition of novel information that favor psychological well-being and autonomy (Schutte and Malouff, 2019).

Precedent research indicates that people identified with the intellectual center report lower levels of aggression than other people characterized with other typological centers (Wagner, 2012; Shameli et al., 2020). It is probable that personality patterns with lower aggression characteristics share a link with Grossarth-Maticek & Eysenck’s (Grossarth-Maticek and Eysenck, 1990) favorable interpersonal reactions to healthy personality, as this personality type is negatively related to aggression in adults (Reyes del Paso and Martínez, 2004).

In the network, the type 6 measure is localized within the associative patterns of the positive coping style (nexus of relationships of types 2, 7, and 9); therefore, it is more likely that university students with greater confidence and positive attitudes will be able to cope and react effectively to various social situations. Likewise, people who identify with the positive style tend to have more enjoyable experiences, are helpful, and are oriented to identify positive qualities in others, even in adverse situations. Causal connections are also shown between the traits of the other coping typological triads: reactive (types 4, 6, and 8) and competence (types 1, 3, and 5) that indicate a balance in the interactive functioning of mental, emotional, and instinctive capacities in coping with social situations linked to healthy behaviors and reactions to psychosocial stress and physical well-being (Hernández and Froján, 2005; Whitfield et al., 2020).

The healthy personality to psychosocial stress demonstrated causal relationships with the three enneatypes of the combative social style (types 3, 7, and 8) which is identified by problem solving, active coping style, assertiveness, and greater identity with the self, compared to the other personal styles of the Enneagram (Nettmann, 2013). The highest magnitude connections with healthy personality were with types 7 and 8, which according to the systematic review of Hook et al. (Hook et al., 2021) evidenced significant positive relationships between the Big Five personality facets of the Enneagram typologies. Eight reviewed investigations presented direct associations between extraversion and type 8, while 10 previous studies reported relationships between the domains of extraversion and openness with type 7 (Hook et al., 2021). Likewise, Hook et al. (Hook et al., 2021) evidenced that a greater preference for extraversion according to the Myers-Briggs Typological Inventory was related to type 7 in six of the seven studies reviewed, and to type 8 in five of the seven investigations. It is possible that those university students identified with these typologies present characteristics such as boldness in social situations, attention to internal experiences, and a greater tendency to experience positive emotions, which reinforce a healthier personality to psychosocial stress, since they are more associated with psychological well-being (Hernández et al., 2007). The findings suggest that people identified with types 7 and 8 present higher levels of self-control and assertiveness, as well as a lower tendency to negative emotions, attachment, and dependence (Wagner, 2012; Araghi et al., 2015).

According to Enneagram theory, the integration of type 7 with its *wings* (types 6 and 8) promotes the development of personal traits of determination, tenacity, responsibility, motivation, reflection, temperance, greater spiritual interest, they are more organized, do not worry about being judged, and are more likely to become leaders (Enneagram, 2019). Research by Roh et al. (Roh et al., 2019) indicated that college students with a higher predominance of type 7 are noted for cognitive empathy, who have a higher degree of consideration of another person’s point of view to appreciate the situation from their perspective (Hojat et al., 2002). Optimism is the main character of type 7 (bridging enneatype) that favors mindfulness and reduces levels of depression, anxiety, and stress. It is also recognized as a protective factor for mental health in the face of the current pandemic (Vos et al., 2021) and is related to a lower risk of mortality according to a recent meta-analysis (Craig et al., 2021). Optimistic individuals are noted for a personality with a positive attitude, more carefree, and flexible to others, these typological characteristics are likely to be the underlying mediating traits influencing the relationship of the more network-centric (loyal) personality and the more emotionally autonomous healthy personality.

The greater relationships in the network of types 2 and 4 linked to the emotional center consolidated feeling-centered characteristics such as emotional awareness and regulation that allow individuals to improve communicative and interpersonal skills in an empathic manner (Shin and Lee, 2020). These individuals pay greater attention to their emotions, have a higher degree of understanding and valuing emotions, clearly identify their own and others’ negative emotions, and then manage them assertively in social relationships. Such characteristics of awareness and self-regulation of emotions are significantly associated with Grossarth-Maticek and Eysenck’s healthy personality (Grossarth-Maticek and Eysenck, 1990). However, in the network, typologies 2 and 4 are only related to the healthy personality through the loyal personality (type 6) characterized by trust and harmony.

## Limitations

This study has some limitations that should be mentioned. First, it should be noted that this is a cross-sectional study; therefore, it cannot be inferred whether a given node causes or is caused by another node to which it is connected due to the use of undirected networks. Second, it is that the cross-sectional edges represent both within- and between-subject effects that cannot be disentangled, i.e., it is not possible to interpret these results at the individual level. Experimental and prospective designs are needed to more rigorously test those assumptions underlying the causal systems perspective for theoretical models of personality. However, the application of this variant of network analysis is important since it guarantees better technical information on the interaction of the variables under study because it estimates the associations after multivariate control of all elements of the system.

Additionally, it is recommended that network analysis be applied in future research that considers various protective and risk factors for mental health during the current pandemic related to personality by type of profession and academic performance in university students for a better interpretation of the results in specific groups and to provide new evidence of personality typologies that are favorable to university education and psychological well-being from the models of complex network systems in diverse sociocultural contexts.

## Conclusion

To conclude, the present research refers the greater importance of the strength of type 6 (number and magnitude of connections) in the network. Type 7 is related to the healthy personality with bridging measures suggesting a direct and indirect associative interconnection pathway of the Enneagram typologies to the healthy personality with psychosocial stress where the characteristics of optimism, curiosity, and psychological flexibility. In this system, higher associations were found between enneatypes 2 and 4, and greater relationships were identified between the personality healthy to psychosocial stress and types 7 and 8. The application of the Bootstrapping method indicates that the relationships and centrality indexes in the network are stable measures.

## Data availability statement

The raw data supporting the conclusions of this article will be made available by the authors, without undue reservation.

## Ethics statement

The studies involving human participants were reviewed and approved by Professional code of Ethics of the Peruvian College of Psychologists. The patients/participants provided their written informed consent to participate in this study.

## Author contributions

CR-V and AB designed the study. JB-C and JVS performed the statistical analysis. CR-V and JS wrote the first draft of the manuscript. All authors contributed to the article and approved the submitted version.

## Funding

Open access funding provided by Universidad Señor de Sipán, Chiclayo, Perú (DIRECTORY RESOLUTION N°015-2022/PD-USS).

## Conflict of interest

The authors declare that the research was conducted in the absence of any commercial or financial relationships that could be construed as a potential conflict of interest.

## Publisher’s note

All claims expressed in this article are solely those of the authors and do not necessarily represent those of their affiliated organizations, or those of the publisher, the editors and the reviewers. Any product that may be evaluated in this article, or claim that may be made by its manufacturer, is not guaranteed or endorsed by the publisher.
